# Tuberculosis and Cellular Metabolism: Insights into the Crosstalk Between Macrophage Immunometabolism and Muscle Dysregulation

**DOI:** 10.3390/ijms27136062

**Published:** 2026-07-06

**Authors:** Mohammed J. A. Haider, Halemah AlSaeed, Fatema Al-Rashed

**Affiliations:** 1 Department of Biological Sciences, Faculty of Science, Kuwait University, Sabah Al-Salem University City, Safat, Kuwait City 13060, Kuwait; mohammed.haider@ku.edu.kw; 2 Immunology and Microbiology Department, Dasman Diabetes Institute, Dasman, Kuwait City 15462, Kuwait; halemah.alsaeed@dasmaninstitute.org

**Keywords:** tuberculosis, *Mycobacterium tuberculosis*, macrophage immunometabolism, Warburg effect, HIF-1α, muscle wasting, cachexia, host-directed therapy, biomarkers, mitochondrial dysfunction

## Abstract

Tuberculosis (TB) remains a leading cause of death from a single infectious agent, and its outcome is shaped not only by *Mycobacterium tuberculosis* (*Mtb*) itself, but also by the host’s metabolic state. This review synthesises current understanding of how *Mtb* reprograms macrophage immunometabolism and how this reprogramming propagates to a systemic level, culminating in skeletal muscle dysregulation and TB-associated cachexia. We describe the molecular mechanisms by which *Mtb* subverts phagosomal maturation, the glycolytic (Warburg-like) switch governed by HIF-1α and accumulation of immunomodulatory tricarboxylic acid cycle intermediates, and the M1/M2 polarisation balance that dictates bacterial containment versus persistence. We then trace the cytokine- and metabolite-mediated circuits (TNF-α, IL-6, IL-1β, lactate, ketone bodies, free fatty acids) that link infected macrophages to ubiquitin–proteasome and autophagy–lysosome-driven muscle proteolysis, mitochondrial dysfunction and oxidative stress. Building on these mechanisms, we propose an immunometabolic and muscle-derived biomarker framework that, although still requiring clinical validation, may offer value for diagnosis, host-response stratification and treatment monitoring, and we discuss host-directed therapeutic strategies that target macrophage metabolism and muscle preservation. By integrating immunity, metabolism and systemic pathology at the molecular level, this work highlights translational opportunities relevant to the host immunity, diagnosis and treatment of tuberculosis.

## 1. Introduction

TB remains a significant global health challenge, with its burden amplified by the complex interplay between host immune responses and systemic metabolic alterations. While the primary pathological hallmark of M. tuberculosis infection lies in the respiratory system, recent advances have illuminated the profound role of cellular metabolism in shaping disease outcomes [1]. This review aims to explore the emerging paradigm of metabolic reprogramming in TB, focusing on the intricate crosstalk between macrophage function and muscle dysregulation.

Central to TB pathophysiology is the macrophage, a pivotal player in host immunity. Following *Mtb* infection, macrophages undergo profound functional and metabolic reprogramming to support pathogen clearance [2]. However, the bacterium has evolved sophisticated mechanisms to subvert these processes, creating a permissive intracellular environment that supports its persistence. The metabolic shifts observed in macrophages, such as enhanced glycolysis [3], impaired mitochondrial function [4], and lipid droplet accumulation, not only modulate immune responses but also have systemic repercussions that extend beyond localised infection [5].

One of the systemic manifestations of TB is muscle wasting, often described as TB-associated cachexia. This debilitating condition reflects profound dysregulation in muscle homeostasis, characterised by increased proteolysis, impaired mitochondrial function, and oxidative stress [6,7]. These changes are thought to be driven, in part, by the inflammatory milieu established by infected macrophages, as well as by systemic metabolic disturbances such as hypercatabolism and altered lipid utilisation [6]. Despite its clinical significance, the mechanisms underpinning this muscle dysregulation and its relationship to macrophage metabolism remain incompletely understood.

Despite this clinical importance, muscle dysfunction in TB has historically attracted far less research attention than pulmonary pathology, for several converging reasons. TB has long been framed primarily as a respiratory disease, with diagnostic and therapeutic priorities centred on bacterial burden, lung damage and transmission, so that systemic and musculoskeletal consequences have often been regarded as secondary downstream effects rather than as targets in their own right. Muscle wasting in TB has also been difficult to disentangle from the general malnutrition and weight loss that accompany chronic infection, leading it to be treated as a nonspecific feature of advanced disease rather than a distinct, mechanistically tractable process [8]. In addition, skeletal muscle is less accessible to routine sampling than sputum or blood, and the tools needed to interrogate muscle immunometabolism in patients have only recently matured. As a result, the molecular crosstalk between infected macrophages and skeletal muscle has remained comparatively unexplored. By placing this crosstalk at its centre, the present review addresses a genuine gap in current understanding and reframes TB-associated muscle dysfunction as an active immunometabolic process rather than a passive consequence of disease.

Given the intertwined nature of immune and metabolic pathways, there is an urgent need to dissect the molecular mechanisms linking macrophage metabolism to muscle dysregulation in TB. This review aims to synthesise current knowledge on these interactions, providing insights into potential therapeutic interventions that address both host immunity and systemic metabolic health. By bridging the gap between immunometabolism and systemic pathology, this work seeks to pave the way for innovative strategies in TB management and treatment.

## 2. Tuberculosis and Macrophage Metabolism

Macrophages play a central role in the pathogenesis of TB, serving as both the primary host cells for *Mtb* and key effectors in the immune response. Upon infection, macrophages initiate a complex cascade of immune and metabolic events aimed at controlling bacterial replication. However, *Mtb* has evolved sophisticated strategies to evade host defences, leading to persistent infection and chronic disease [9]. Understanding the metabolic and functional dynamics of macrophages in TB is critical to uncovering new therapeutic targets.

### 2.1. Role of Macrophages in TB Pathogenesis

Upon entry into the host airways, *Mtb* infects alveolar macrophages (AMs), followed by the subsequent invasion of recruited interstitial macrophages. *Mtb* manipulates host intracellular signalling pathways to evade immune clearance and establish a niche for intracellular survival. Uptake of *Mtb* occurs through various receptors, including complement receptors (CR3, CR4) [10], C-type lectin receptors such as Dectin-1 [11], and scavenger receptors [12], leading to the recruitment of Src family protein tyrosine kinases (PTKs) and the activation of phospholipase D (PLD) [13]. This activation increases phosphatidic acid (PA) production, a crucial lipid involved in membrane remodelling and vesicular trafficking, and can enhance the activation of NADPH oxidase, a critical component of the respiratory burst [14]. However, despite these early signalling events, *Mtb* subverts normal phagosomal maturation to avoid destruction within alveolar macrophages.

One of the primary evasion strategies utilised by *Mtb* is the inhibition of phagosomal acidification; this is essential for activating lysosomal hydrolases and generating an antimicrobial environment. In contrast to typical phagosomes that undergo progressive acidification upon fusion with lysosomes, mycobacterial phagosomes exhibit a significantly reduced acidification profile [15,16]. This impairment is attributed to the defective recruitment and assembly of vacuolar H^+^-ATPase (v-ATPase) on the phagosomal membrane [17,18], preventing efficient proton transport into the lumen. Instead of reaching the acidic pH of ~4.5 typically seen in mature phagolysosomes, *Mtb*-containing phagosomes stabilise at a neutral to mildly acidic pH (~6.3–6.5), which is insufficient for optimal enzymatic degradation of bacterial components [19,20]. The reduced recruitment of v-ATPase has been linked to mycobacterial lipid components, including trehalose dimycolate (TDM) and phosphatidylinositol mannosides (PIMs), which interfere with phagosomal maturation by altering lipid raft organisation and impairing vesicular trafficking.

Additionally, *Mtb* manipulates alternative proton sources to further prevent phagosomal acidification. The Na^+^/H^+^ exchanger, which is incorporated into *Mtb*-containing phagosomes, appears to have a negligible impact on acidification due to the dependency on luminal Na^+^ concentrations. Interestingly, inhibition of v-ATPase activity further attenuates the mild acidification (~1 pH unit) of mycobacterial phagosomes, suggesting that despite its limited recruitment, v-ATPase remains a restricted but direct source of protons for these compartments. Experimental activation of v-ATPase with exogenous cofactors has been shown to partially restore phagosomal acidification, emphasising the active role *Mtb* plays in preventing full v-ATPase functionality [16].

Beyond inhibiting acidification, *Mtb*-containing phagosomes also evade fusion with lysosomes, further protecting the bacterium from degradation. This blockade is mediated by *Mtb*-secreted effectors, including acid phosphatase (SapM), that depletes phosphatidylinositol 3-phosphate (PI3P), a critical lipid marker required for phagosomal maturation [21]. Another effector, PtpA, dephosphorylates host proteins involved in vesicular trafficking, further preventing phagosome–lysosome fusion [22]. *Mtb* also exploits host Rab GTPases, particularly Rab5 and Rab7, which regulate endosomal maturation. While Rab5 is transiently recruited to *Mtb* phagosomes, Rab7 recruitment is actively inhibited, preventing the transition to a degradative lysosomal compartment [23].

### 2.2. Metabolic Reprogramming in Infected Macrophages

**Figure 1:**
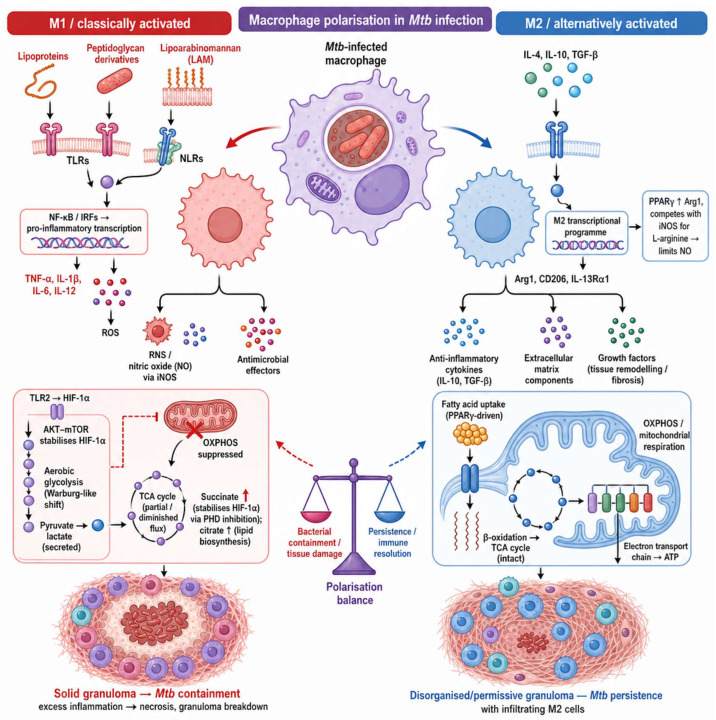
Macrophage polarisation and immunometabolic reprogramming during *Mycobacterium tuberculosis* infection. The *Mtb*-infected macrophage polarises between two opposing programmes. The classically activated M1 programme (red; left), driven by TLR/NLR sensing of mycobacterial components through NF-κB and IRFs, produces pro-inflammatory cytokines (TNF-α, IL-1β, IL-6, IL-12), ROS and NO, and undergoes a HIF-1α-governed Warburg-like shift to aerobic glycolysis with lactate secretion, partial TCA flux and suppressed OXPHOS; accumulating succinate and citrate act as immunomodulatory signals. This programme favours granuloma formation and bacterial containment. The alternatively activated M2 programme (blue; right), driven by IL-4, IL-10 and TGF-β, supports tissue remodelling and relies on fatty acid oxidation, an intact TCA cycle and OXPHOS, favouring a permissive granuloma and *Mtb* persistence. The central balance denotes the M1–M2 equilibrium dictating containment versus persistence. Figure created in Illustrae.co.

The impact of *Mtb* also extends to shaping the overall immune milieu. Macrophage polarisation plays a crucial role in this process, influencing both bacterial clearance and disease progression. Upon infection with *Mtb*, macrophages can adopt either a pro-inflammatory (M1) or an anti-inflammatory (M2) phenotype, each characterised by distinct functional and metabolic programmes [24] (Figure 1).

The dynamic balance between these polarisation states determines the host’s ability to control infection while minimising tissue damage.

M1 macrophages, also known as classically activated macrophages, represent the first line of innate immune defence against *Mtb*. Their activation is driven by pattern recognition receptor (PRR) signalling, particularly through toll-like receptors (TLRs) and nucleotide-binding oligomerisation domain (NOD)-like receptors (NLRs), which recognise mycobacterial components such as lipoproteins, lipoarabinomannan (LAM), and peptidoglycan derivatives [12]. These signals activate nuclear factor kappa B (NF-κB) and interferon regulatory factors (IRFs), leading to the transcription of pro-inflammatory cytokines, including TNF-α, IL-1β, IL-6, and IL-12 [25,26]. M1 macrophages also secrete reactive oxygen species (ROS) and reactive nitrogen species (RNS), particularly nitric oxide (NO), through inducible nitric oxide synthase (iNOS), contributing to antimicrobial activity [27]. However, *Mtb* has evolved mechanisms to counteract these defences, including the secretion of superoxide dismutase (SodA) and catalase–peroxidase (KatG), which neutralise ROS, and the production of PtpA, which inhibits phagosomal acidification and autophagic pathways.

Metabolically, M1 macrophages undergo a profound shift towards aerobic glycolysis, a phenomenon similar to the Warburg effect observed in tumour cells. This metabolic reprogramming allows for rapid ATP production to sustain their inflammatory functions while also supporting biosynthetic pathways necessary for cytokine production and antimicrobial activity [28,29]. During *Mtb* infection, recruited monocyte-derived macrophages predominantly adopt this glycolytic phenotype, relying on glucose fermentation even in the presence of oxygen. The central regulator of this metabolic switch is hypoxia-inducible factor 1-alpha (HIF-1α), a transcription factor that upregulates key glycolytic enzymes, facilitating the preferential conversion of glucose to lactate rather than channelling it through the tricarboxylic acid (TCA) cycle [30]. HIF-1α expression is induced following TLR activation, particularly through TLR2, and is further amplified by pro-inflammatory mediators such as IL-1β, TNF-α, and NO [30,31]. These inflammatory signals converge on NF-κB, which enhances HIF-1α transcription, thereby sustaining glycolytic flux. Additionally, activation of the protein kinase B (AKT)-mechanistic target of rapamycin (mTOR) pathway further supports HIF-1α stabilisation and glycolytic commitment [4].

As glycolysis becomes the dominant metabolic pathway in M1 macrophages, the fate of pyruvate shifts from its mitochondrial conversion to acetyl coenzyme A (CoA) for oxidative phosphorylation (OXPHOS) to its reduction into lactate, which is secreted extracellularly [32]. This redirection results in a diminished supply of acetyl-CoA, limiting the entry of substrates into the TCA cycle. Consequently, critical OXPHOS enzymes are downregulated, further reinforcing the reliance on glycolysis for energy production [33]. However, despite the suppression of mitochondrial respiration, immunomodulatory TCA cycle intermediates, such as succinate and citrate, accumulate. Succinate plays a crucial role in stabilising HIF-1α by inhibiting prolyl hydroxylases (PHDs), which would otherwise target HIF-1α for degradation. This sustains an inflammatory state by driving the expression of glycolytic genes, as well as pro-inflammatory cytokines such as IL-1β. Additionally, citrate accumulation supports lipid biosynthesis for the production of inflammatory mediators, further shaping the macrophage response to *Mtb* infection [34].

In contrast, M2 macrophages, or alternatively activated macrophages, are associated with tissue repair and immune regulation. Their activation is induced by anti-inflammatory cytokines such as IL-4, IL-10, and transforming growth factor-beta (TGF-β), which drive the expression of markers like arginase-1 (Arg1), CD206, and IL-13 receptor alpha 1 (IL-13Rα1) [35,36]. Unlike M1 macrophages, M2 macrophages rely on oxidative phosphorylation and fatty acid metabolism to sustain their functions, utilising pathways such as β-oxidation and mitochondrial respiration [37]. This metabolic adaptation supports their ability to secrete extracellular matrix components, growth factors, and anti-inflammatory cytokines that promote tissue remodelling and fibrosis [38,39].

The polarisation balance between M1 and M2 macrophages significantly influences TB pathogenesis. A robust M1 response is essential for early containment of *Mtb*, but excessive inflammation can lead to collateral tissue damage, necrosis, and granuloma breakdown, contributing to disease progression. On the other hand, an excessive M2 response, often observed in chronic or latent TB, favours bacterial persistence by suppressing immune activation and facilitating intracellular survival.

One striking example of this is the impact of M2-associated cytokines, particularly IL-10 and TGF-β, in shaping the immune response to *Mtb*. IL-10, a hallmark cytokine secreted by M2 macrophages, suppresses antimycobacterial immunity by inhibiting pro-inflammatory cytokine production and dampening T cell activation, ultimately creating a permissive environment for *Mtb* survival [40]. Similarly, TGF-β, another immunosuppressive cytokine produced by M2 macrophages, has been shown to inhibit IFN-γ-mediated responses from T cells, thereby impairing the host’s ability to mount an effective immune defence. In both human and murine models of TB, the deletion of TGF-β signalling leads to enhanced cytotoxic T cell responses within granulomas and a significant reduction in bacterial burden [41,42].

**Figure 2:**
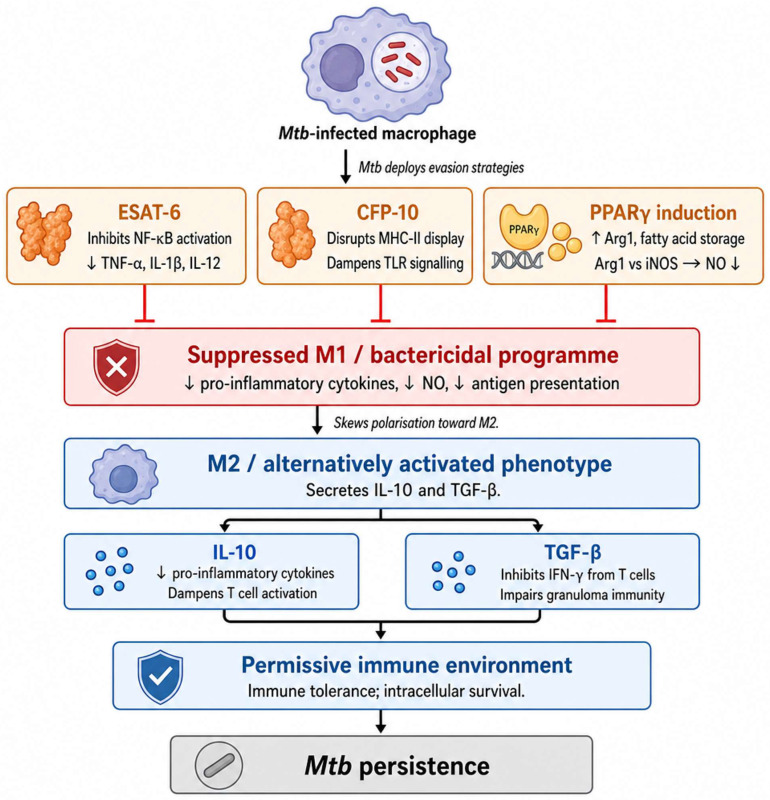
*Mycobacterium tuberculosis* subverts macrophage immunity to suppress the M1 programme and drive M2-associated persistence. *Mtb* deploys parallel evasion strategies that converge on the bactericidal M1 response: ESAT-6 inhibits NF-κB and pro-inflammatory cytokine production; CFP-10 disrupts MHC-II display and TLR signalling; and *Mtb*-induced PPARγ promotes arginase-1 expression, which competes with iNOS for L-arginine and limits NO. Suppression of M1 activation skews polarisation towards an M2 phenotype secreting IL-10 and TGF-β, which further dampen pro-inflammatory responses, T-cell activation and granuloma immunity, establishing a permissive environment that favours bacterial survival and persistence. Colour coding: amber, *Mtb*-derived virulence factors; red, the suppressed M1 programme; blue, the M2 evasion axis. Arrows indicate activation, suppression or skewing of macrophage programmes by *Mtb*-derived factors. Figure created in Illustrae.co.

Another form of M2-mediated immune evasion employed by *Mtb* is the secretion of virulence factors that actively suppress M1 polarisation while enhancing M2-like characteristics. In particular, virulence factors such as early secreted antigenic target 6 kDa (ESAT-6) and culture filtrate protein 10 (CFP-10) modulate macrophage signalling pathways to suppress M1 polarisation while promoting the induction of M2-like characteristics. ESAT-6 has been shown to inhibit NF-κB activation, thereby reducing the production of pro-inflammatory cytokines such as TNF-α, IL-1β, and IL-12, which are essential for activating antimicrobial responses. Meanwhile, CFP-10 disrupts antigen presentation by interfering with major histocompatibility complex class II (MHC-II) expression, which subsequently dampens TLR-mediated signalling, further skewing macrophages away from a bactericidal phenotype. This immune modulation not only limits *Mtb* clearance but also contributes to immune tolerance, allowing the pathogen to persist within host cells. A further strategy by which *Mtb* enhances the M2 response is through the induction of peroxisome proliferator-activated receptor gamma (PPARγ), a key regulator of lipid metabolism that promotes fatty acid uptake and storage while simultaneously inhibiting inflammatory responses [43]. Activation of PPARγ leads to the upregulation of arginase-1 (Arg1), which competes with iNOS for the common substrate L-arginine. By favouring arginase activity over iNOS, *Mtb* effectively limits the production of nitric oxide (NO), a critical antimicrobial molecule required for bacterial clearance [44,45] (Figure 2).

It is important to recognise that the M1 and M2 designations represent simplified extremes of a continuous and highly dynamic spectrum rather than fixed, stable states. During the course of *Mtb* infection, individual macrophages transition between metabolic and activation programmes as the local environment changes, and the dominant phenotype shifts with the stage of infection, from early innate control through granuloma formation to chronic or latent disease. Considerable heterogeneity also exists within a single granuloma: macrophages occupying different spatial niches, such as the hypoxic caseous centre versus the better-perfused periphery, experience distinct oxygen and nutrient availability and adopt correspondingly different metabolic states [46,47]. Foamy, epithelioid and recently recruited monocyte-derived macrophages coexist within the same lesion, each with a distinct immunometabolic profile. The M1/M2 framework used throughout this review should therefore be understood as a conceptual scaffold that captures the dominant polarising influences at a given point, rather than as evidence of a single stable phenotype; in reality, macrophage metabolism in TB is plastic, spatially heterogeneous and continuously remodelled over the disease course.

## 3. Systemic Metabolic Dysregulation in TB

*Mtb* is also able to reside and thrive in a number of other non-myeloid cellular niches, such as respiratory epithelial cells, muscle cells, and adipocytes, where it can evade immune detection and persist in a quiescent state. These alternative reservoirs provide *Mtb* with a protective environment, shielding it from immune-mediated clearance while also serving as potential sources for reactivation and dissemination under conditions of immune suppression. To this end, the pathology of TB extends beyond a localised infectious disease and also manifests as a condition with profound systemic metabolic consequences. Among these, the most debilitating is the development of cachexia, a syndrome of severe weight loss and muscle wasting that significantly impacts patient morbidity and mortality [48]. Understanding the metabolic underpinnings of this systemic dysregulation is crucial for developing effective interventions to improve outcomes in TB patients.

### 3.1. Energy Balance and Cachexia

Cachexia is a hallmark of TB, characterised by a dramatic loss of fat and muscle mass that occurs despite adequate nutritional intake in many cases. This condition reflects a profound imbalance in energy metabolism, driven by both increased energy expenditure and impaired nutrient utilisation. Muscle protein degradation and adipose tissue lipolysis are central features, leading to a decline in physical function and increased susceptibility to secondary complications.

Systemic inflammation is a key driver of the catabolic processes underlying cachexia in TB. Pro-inflammatory cytokines, such as TNF-α, IL-1β, and IL-6, are elevated during TB infection and play a central role in promoting muscle protein breakdown and fat mobilisation. These cytokines act on multiple metabolic pathways, increasing energy expenditure and reducing anabolic signalling, further exacerbating tissue wasting. Additionally, the inflammatory milieu disrupts mitochondrial function in muscle cells, contributing to reduced energy production and enhanced oxidative stress.

The interplay between systemic inflammation and metabolic dysregulation highlights the complexity of TB-associated cachexia. By linking localised immune responses with widespread metabolic effects, this phenomenon underscores the need for comprehensive therapeutic approaches that target both the inflammatory and metabolic components of the disease. Addressing these interconnected processes holds promise for mitigating the systemic burden of TB and improving patient outcomes.

### 3.2. Inflammatory Cytokines and Metabolic Alterations

Inflammatory cytokines play a central role in the systemic metabolic dysregulation observed in tuberculosis (TB). These signalling molecules, primarily produced by immune cells, act as mediators of the chronic inflammation characteristic of TB. Their impact extends beyond immune activation, driving significant alterations in host metabolic pathways and contributing to disease-associated complications such as cachexia and energy imbalance.

Key pro-inflammatory cytokines, including tumour necrosis factor-alpha (TNF-α) and interleukin-6 (IL-6), are major contributors to the systemic inflammation observed in TB. TNF-α, a hallmark cytokine in TB, promotes lipolysis and proteolysis, leading to the depletion of adipose tissue and skeletal muscle mass [49]. Simultaneously, IL-6 enhances hepatic gluconeogenesis and induces acute-phase protein synthesis, further disrupting energy homeostasis [50]. These cytokines act in concert to amplify metabolic stress, impair anabolic processes, and perpetuate a catabolic state.

The crosstalk between cytokine signalling and metabolic pathways is a key mechanism underlying these alterations. For example, TNF-α and IL-6 interact with intracellular pathways such as the NF-κB and Janus kinase/signal transducer and activator of transcription (JAK/STAT) pathways, which regulate both immune responses and metabolic processes. These interactions lead to downstream effects, such as increased mitochondrial dysfunction, ROS production, and altered lipid metabolism. Additionally, cytokine-mediated insulin resistance disrupts glucose uptake and utilisation in peripheral tissues, further exacerbating systemic energy imbalances [51].

The interplay between inflammatory cytokines and metabolic pathways creates a vicious cycle in TB, where inflammation drives metabolic dysfunction, which, in turn, amplifies immune dysregulation. Addressing this cytokine–metabolism axis presents an opportunity for therapeutic interventions aimed at mitigating the systemic effects of TB while enhancing host resilience and recovery.

## 4. Muscle Dysregulation in TB

Muscle dysregulation is a defining feature of TB-associated cachexia, characterised by profound muscle wasting and weakness. This condition arises from a complex interplay of catabolic and anabolic imbalances, which disrupt muscle protein homeostasis. Understanding the molecular pathways underlying muscle degradation is essential for developing targeted interventions to preserve muscle mass and function in TB patients.

### 4.1. Pathways of Muscle Wasting

It should be noted that much of the molecular detail of these pathways derives from cancer cachexia, sepsis, disuse atrophy and other chronic inflammatory wasting models rather than from direct study of skeletal muscle in TB. The ubiquitin–proteasome and autophagy–lysosome pathways, the roles of atrogin-1 and MuRF1, and the suppression of mTOR-dependent synthesis are well established as general mechanisms of inflammatory muscle wasting, and the cytokine drivers implicated (TNF-α, IL-6, IL-1β) are demonstrably elevated in TB; however, the direct demonstration of these specific catabolic pathways within the skeletal muscle of patients with TB remains limited. The mechanisms described in this section should therefore be read as a well-supported framework extrapolated to TB, the TB-specific validation of which is still emerging.

Two primary pathways are thought to drive muscle protein degradation in TB: the ubiquitin–proteasome system (UPS) and the autophagy–lysosome pathway. The UPS is the principal mechanism of muscle protein breakdown, involving the tagging of proteins with ubiquitin and their subsequent degradation by the proteasome. In TB, systemic inflammation and elevated levels of pro-inflammatory cytokines, such as TNF-α and IL-6, activate this pathway. This leads to increased expression of muscle-specific E3 ubiquitin ligases, such as atrogin-1 and MuRF1, which target structural and contractile proteins for degradation [52].

The autophagy–lysosome pathway complements the UPS by degrading cellular organelles and larger protein complexes. In TB, enhanced autophagic activity in muscle cells is thought to contribute to further protein loss. While autophagy is a normal cellular process for maintaining homeostasis, its dysregulation during chronic inflammation exacerbates muscle atrophy [53,54].

In addition to increased protein degradation, muscle wasting in TB is driven by the dysregulation of anabolic signalling pathways, particularly the mechanistic target of rapamycin (mTOR) pathway. mTOR is a central regulator of muscle protein synthesis, responding to nutrient availability, energy status, and growth factors. In TB, inflammatory cytokines and metabolic stress impair mTOR signalling, leading to reduced protein synthesis and an imbalance favouring catabolism [55]. This inhibition of anabolic pathways further accelerates muscle loss, compounding the effects of enhanced proteolysis.

**Figure 3:**
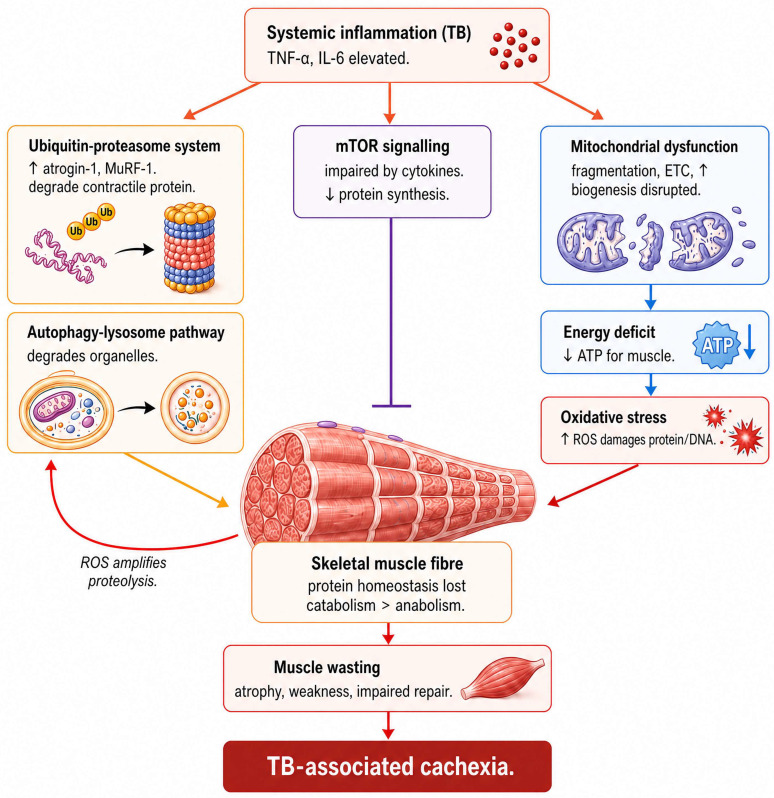
Mechanisms of skeletal muscle dysregulation in TB-associated cachexia. Systemic inflammation (elevated TNF-α and IL-6) drives muscle protein loss through three converging routes: proteasomal degradation via cytokine-induced E3 ubiquitin ligases atrogin-1 and MuRF1, together with the autophagy–lysosome pathway; impaired mTOR signalling that reduces protein synthesis; and mitochondrial dysfunction producing an energy deficit and excess ROS, which further amplifies proteolysis in a self-reinforcing catabolic cycle. These pathways converge on the muscle fibre, where catabolism exceeds anabolism, leading to muscle wasting and ultimately TB-associated cachexia. Colour coding: amber, proteolytic pathways; purple, the mTOR anabolic axis; blue, mitochondrial compromise; red, inflammatory input, oxidative stress and clinical outcome. Arrows indicate activation or promotion of the catabolic pathways depicted. Figure created in Illustrae.co.

The dual activation of proteolytic pathways and suppression of anabolic signalling creates a severe catabolic state in TB patients (Figure 3). Understanding these mechanisms highlights the need for therapies that target both proteasomal activity and anabolic dysregulation, offering potential strategies to mitigate muscle wasting and improve patient outcomes.

### 4.2. Metabolic Changes in Muscle During TB

Muscle metabolic alterations are a significant aspect of tuberculosis (TB)-associated muscle wasting, driven by systemic inflammation and catabolic stress. In TB, these metabolic disruptions contribute to mitochondrial dysfunction, impaired energy production, and oxidative stress, all of which exacerbate muscle degradation and compromise physical function.

Mitochondrial dysfunction is a hallmark of metabolic dysregulation in muscle during TB. Mitochondria play a central role in energy production through oxidative phosphorylation, and their impairment leads to reduced ATP availability, limiting the energy required for maintaining muscle integrity and function. Pro-inflammatory cytokines such as TNF-α and IL-6, which are elevated in TB, disrupt mitochondrial biogenesis and dynamics, leading to fragmentation and decreased efficiency of the electron transport chain [56]. At the molecular level, evidence from cancer cachexia and other inflammatory wasting conditions indicates that these cytokines, together with reactive oxygen species, suppress the master regulator of mitochondrial biogenesis, peroxisome proliferator-activated receptor-γ coactivator-1α (PGC-1α), and its downstream effectors nuclear respiratory factor-1 (NRF-1) and mitochondrial transcription factor A (TFAM), thereby reducing the synthesis of new mitochondria [57]. In parallel, mitochondrial quality control becomes dysregulated: the balance of mitochondrial fusion (governed by mitofusins MFN1/2 and OPA1) and fission (governed by DRP1) shifts towards fragmentation, while clearance of damaged mitochondria through PINK1/Parkin-mediated mitophagy is altered, leading to the accumulation of dysfunctional organelles [56]. This results in reduced energy output and contributes to the energy deficit observed in TB-associated cachexia. Whether these specific regulatory changes occur in the skeletal muscle of patients with TB remains to be directly established, but they represent a well-characterised mechanism in comparable catabolic states.

In addition to compromised energy production, mitochondrial dysfunction in TB is closely linked to increased oxidative stress. Dysfunctional mitochondria generate excess ROS, which can damage cellular components such as proteins, lipids, and DNA. Elevated ROS levels further activate proteolytic pathways, including the ubiquitin–proteasome system and autophagy–lysosome pathways, accelerating muscle protein degradation. The accumulation of oxidative damage also impairs muscle repair and regeneration, perpetuating the cycle of muscle wasting.

The interplay between mitochondrial dysfunction, energy deficit, and oxidative stress creates a metabolic environment that favours catabolism over anabolism in TB. These metabolic changes underscore the importance of therapeutic approaches that target mitochondrial health and oxidative balance to preserve muscle mass and function. By addressing the metabolic roots of muscle dysregulation, such interventions could improve outcomes for TB patients and reduce the burden of cachexia.

## 5. Linking Macrophage Metabolism to Muscle Dysregulation

These mechanistic considerations are reflected in consistent clinical observations of impaired nutritional status and altered body composition in patients with TB. Low body mass index (BMI) is common at diagnosis and is both a risk factor for progression to active disease and a predictor of adverse treatment outcomes and mortality [58,59]. Weight loss is a cardinal feature of active TB, and body composition studies show that this reflects loss of both fat mass and, importantly, fat-free (lean) mass, with low skeletal muscle mass increasingly recognised as a distinct component of TB-associated malnutrition. Notably, the catabolic drive of TB can blunt the anabolic response to nutrition: in patients with multidrug-resistant TB, body weight and fat-free mass decline over the course of treatment despite increased macronutrient intake, consistent with an inflammation-driven catabolic state rather than simple undernutrition. Lean tissue mass, more than weight or BMI alone, correlates with physical functioning and survival, underscoring the clinical importance of the muscle-wasting mechanisms discussed below.

**Figure 4:**
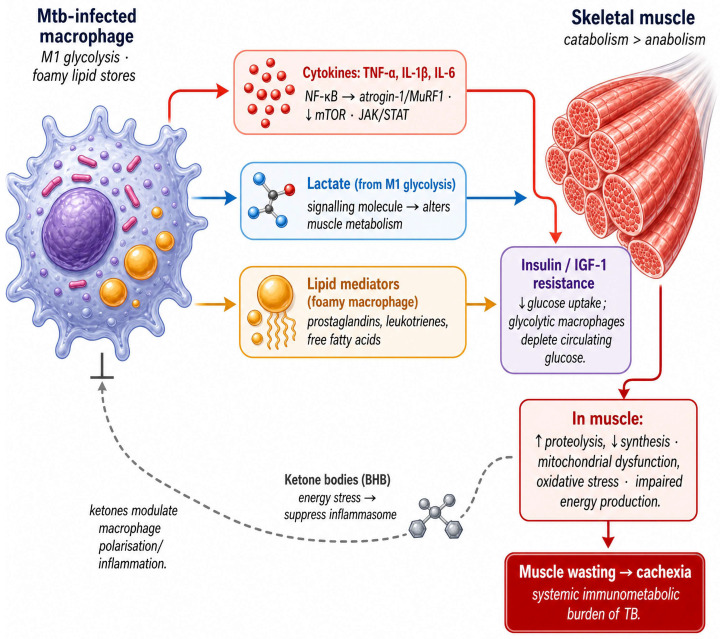
Immunometabolic crosstalk linking macrophage metabolism to skeletal muscle dysregulation in TB. Metabolic reprogramming of the *Mtb*-infected macrophage generates mediators that act systemically on muscle to favour catabolism. Pro-inflammatory cytokines (TNF-α, IL-1β, IL-6) activate atrogin-1 and MuRF1 and suppress mTOR-dependent synthesis; macrophage-derived lactate acts as a signalling molecule altering muscle metabolism rather than as a mere by-product; lipid mediators from foamy macrophages modulate mitochondrial function; and cytokine-driven insulin and IGF-1 resistance reduces muscle glucose uptake. These inputs increase proteolysis, reduce synthesis and promote mitochondrial dysfunction, culminating in muscle wasting and cachexia. The relationship is bidirectional: ketone bodies generated under energy stress feed back to suppress macrophage inflammasome activation (grey dashed connector). Colour coding: coral, cytokine signalling; blue, lactate; amber, lipid mediators; purple, insulin/IGF-1 resistance; red, muscle consequences and outcome. Arrows indicate the direction of mediator signalling from macrophage to muscle; the grey dashed arrow denotes ketone-body feedback to the macrophage. Figure created in Illustrae.co.

The systemic effects of TB extend beyond localised infection, with macrophage metabolism playing a central role in driving muscle dysregulation. Macrophages, as key immune effectors, release pro-inflammatory cytokines that mediate catabolic processes in muscle tissue. Understanding the cytokine-mediated mechanisms linking macrophage activity to muscle atrophy is essential to addressing the systemic burden of TB-associated cachexia. Taken together, these observations connect a localised immune event to a systemic catabolic state through a continuous mechanistic chain. Within the infected lung, macrophage metabolic reprogramming towards aerobic glycolysis and lipid accumulation sustains a pro-inflammatory secretory phenotype, releasing TNF-α, IL-1β and IL-6 into the circulation. These cytokines act at a distance on skeletal muscle, where they activate the ubiquitin–proteasome and autophagy–lysosome pathways while suppressing mTOR-dependent protein synthesis, and concurrently drive insulin resistance, mitochondrial dysfunction and oxidative stress that impair muscle energy metabolism. The net effect is a shift in muscle from anabolism towards catabolism, manifesting as progressive muscle loss and, ultimately, TB-associated cachexia. The sections that follow examine each node of this macrophage-to-muscle axis in turn, and the integrated circuit is summarised in Figure 4.

### 5.1. Cytokine Mediation

Macrophage-derived cytokines, particularly IL-1β, IL-6, and TNF-α, are considered critical drivers of muscle atrophy, largely on the basis of evidence from cancer and other chronic inflammatory cachexias, with TB-specific data still emerging. These cytokines are upregulated during *Mtb* infection as part of the host immune response and act on muscle tissue to induce catabolic changes. TNF-α, for example, activates NF-κB signalling, promoting the expression of muscle-specific E3 ubiquitin ligases such as atrogin-1 and MuRF1, which are central to proteasomal degradation of muscle proteins [52]. Similarly, IL-1β and IL-6 contribute to the suppression of anabolic signalling pathways, including mTOR, further tipping the balance towards muscle breakdown [55].

Mechanistically, cytokines mediate muscle catabolism by disrupting intracellular signalling pathways in myocytes. TNF-α and IL-6 interfere with insulin and insulin-like growth factor-1 (IGF-1) signalling, leading to insulin resistance and reduced activation of mTOR, a key regulator of protein synthesis [51]. Additionally, IL-6 promotes activation of the JAK/STAT pathway, which contributes to metabolic dysregulation and muscle wasting [55]. These cytokines also exacerbate mitochondrial dysfunction and oxidative stress in muscle cells, further impairing energy production and amplifying catabolic processes.

The interplay between macrophage-derived cytokines and muscle metabolism highlights a complex, bidirectional relationship in which systemic inflammation driven by infected macrophages leads to profound metabolic consequences in muscle tissue. Addressing these inflammatory mechanisms presents an opportunity to mitigate muscle atrophy in TB patients and reduce the overall systemic burden of the disease. Targeted therapeutic approaches that disrupt this cytokine-mediated crosstalk could play a pivotal role in managing TB-associated cachexia.

### 5.2. Systemic Metabolic Effects

The metabolic reprogramming of macrophages during TB infection has far-reaching consequences that extend beyond localised immune responses. These changes significantly influence systemic glucose and lipid metabolism, creating a metabolic environment that exacerbates tissue dysfunction, including muscle dysregulation. Lipid mediators play a central role in linking macrophage activity to systemic metabolic outcomes, impacting both immune function and muscle health.

Macrophage metabolic changes during *Mtb* infection disrupt systemic glucose homeostasis. The glycolytic shift observed in activated macrophages increases their local glucose consumption to support inflammatory responses. While activated immune cells are unlikely to deplete systemic glucose substantially, this heightened demand may contribute to local competition for glucose within the infected tissue microenvironment, and the broader inflammatory state influences glucose availability to peripheral tissues such as muscle. Furthermore, elevated levels of TNF-α and IL-6 drive cytokine-mediated insulin resistance in peripheral tissues, including skeletal muscle.

Systemic inflammation impairs insulin signalling in skeletal muscle through several converging mechanisms. Under normal conditions, insulin stimulates tyrosine phosphorylation of insulin receptor substrate-1 (IRS-1), activating the phosphatidylinositol 3-kinase (PI3K)-Akt pathway and promoting translocation of the glucose transporter GLUT4 to the muscle cell membrane for glucose uptake. Pro-inflammatory cytokines disrupt this cascade. TNF-α activates the serine/threonine kinases c-Jun N-terminal kinase (JNK) and inhibitor of κB kinase (IKKβ), which phosphorylate IRS-1 on serine residues (notably Ser307); this serine phosphorylation impairs the normal insulin-stimulated tyrosine phosphorylation of IRS-1, blunting downstream PI3K-Akt activation and reducing GLUT4-mediated glucose uptake [60]. IL-6 contributes by signalling through the JAK/STAT pathway and inducing suppressor of cytokine signalling-3 (SOCS3), which further downregulates IRS-1 and GLUT4 expression [61]. The net effect in muscle is reduced glucose disposal, an energy deficit, and a metabolic environment that compounds the catabolic drive of cachexia. Insulin resistance is known to arise from multiple inflammatory and metabolic insults [62], and in TB, the sustained cytokine load provides a plausible inflammatory driver of the impaired muscle glucose handling observed during active disease.

Lipid metabolism is another critical aspect of macrophage-driven systemic metabolic dysregulation. Infected macrophages often accumulate lipid droplets, creating a phenotype known as foamy macrophages. These lipid reservoirs serve as both an energy source for *Mtb* and a signalling platform for the production of lipid mediators, such as prostaglandins and leukotrienes. These bioactive lipids influence both local immune responses and systemic metabolic processes. For instance, lipid mediators are thought to modulate muscle mitochondrial function and inflammatory signalling, contributing to oxidative stress and protein degradation in muscle tissue.

Additionally, systemic alterations in lipid profiles during TB, including increased circulating free fatty acids and altered lipoprotein composition, reflect the metabolic consequences of macrophage lipid dysregulation. These changes not only fuel inflammatory pathways but also disrupt lipid utilisation in muscle, impairing energy production and exacerbating muscle wasting.

The systemic metabolic effects of macrophage reprogramming highlight the interconnectedness of immune and metabolic systems in TB. Lipid mediators, in particular, serve as crucial links between macrophage metabolism and muscle dysfunction, underscoring the importance of targeting these pathways to mitigate the broader consequences of TB. Therapeutic interventions that restore metabolic balance may hold promise in addressing both immune dysfunction and the systemic complications of TB.

### 5.3. Immunometabolic Circuits

The interplay between macrophage polarisation states and systemic metabolism in TB creates intricate immunometabolic circuits that influence disease progression and systemic outcomes. The dynamic shifts between macrophage activation states, namely, pro-inflammatory (M1) and anti-inflammatory (M2), have profound implications for metabolic homeostasis, mediated in part by metabolites such as lactate and ketone bodies, which act as signalling molecules and energy sources.

Macrophage polarisation directly affects systemic metabolic outcomes. M1 macrophages, which dominate during the initial immune response to *Mtb*, rely heavily on glycolysis, producing high levels of lactate as a byproduct. This lactate can accumulate in the systemic circulation, where it acts as a signalling molecule that alters muscle metabolism and mitochondrial function rather than serving merely as a metabolic byproduct [32]. Sustained exposure to elevated lactate, alongside the broader inflammatory milieu, may reprogramme muscle energy metabolism in ways that favour catabolism over anabolism. In contrast, M2 macrophages, which are associated with tissue repair and immune resolution, utilise oxidative phosphorylation and fatty acid oxidation. However, excessive M2 polarisation may contribute to lipid storage and dysregulated lipid metabolism, creating systemic imbalances that further impact muscle function.

Crosstalk through metabolites such as lactate and ketone bodies exemplifies the bidirectional communication between macrophages and peripheral tissues. Lactate, in addition to being an energy substrate, acts as a signalling molecule that modulates inflammatory responses and metabolic pathways in both immune and non-immune cells. Similarly, ketone bodies, which are produced during states of high lipid oxidation or energy stress, can influence macrophage polarisation and function. For instance, β-hydroxybutyrate (BHB), a primary ketone body, has been shown to suppress inflammasome activation in macrophages, potentially modulating the systemic inflammatory response in TB [63].

These immunometabolic circuits underscore the interconnectedness of macrophage activity, systemic metabolic regulation, and muscle integrity in TB. Understanding the signalling roles of metabolites like lactate and ketone bodies offers valuable insights into potential therapeutic interventions that target these pathways. By modulating the balance of macrophage polarisation and systemic metabolic flux, it may be possible to mitigate the systemic consequences of TB and improve patient outcomes.

It should be emphasised that, whereas the glycolytic reprogramming of infected macrophages and cytokine-driven proteolysis are well established, the systemic signalling roles proposed for metabolites such as lactate and ketone bodies in skeletal muscle remain largely hypothesis-generating and are extrapolated in part from non-TB models; direct experimental confirmation in TB is required.

## 6. Immunometabolic and Muscle-Derived Biomarkers: Implications for Diagnosis and Disease Monitoring

The mechanistic links described above position the host immunometabolic and muscle compartments not only as drivers of pathology, but also as a potential source of molecular readouts relevant to the diagnosis, staging and monitoring of tuberculosis. Because *Mtb* actively reshapes macrophage metabolism and, through systemic mediators, skeletal muscle homeostasis, the resulting molecular changes are candidate correlates of infection status, host-response quality and treatment trajectory. These candidate signatures, together with their biological source, proposed clinical application and current level of evidence, are summarised in Table 1.

### 6.1. Metabolic Signatures as Diagnostic and Prognostic Indicators 

The Warburg-like glycolytic shift in infected and bystander myeloid cells generates measurable metabolic intermediates that may report on host-response intensity. Accumulation of lactate, succinate and citrate, together with HIF-1α-dependent transcriptional programmes, reflects the degree of pro-inflammatory macrophage activation during *Mtb* infection [30,32]. Circulating or cell-associated profiles of these intermediates, and of the pro-inflammatory cytokines that sustain them (TNF-α, IL-6, IL-1β), may help distinguish protective from pathology-promoting responses and could complement microbiological confirmation. Lipid-laden foamy macrophages and the associated shift in circulating free fatty acids and lipoprotein composition represent a further metabolic axis with biomarker potential, given their consistent association with bacterial persistence and disease activity [41]. Importantly, these readouts should be interpreted as host-response indicators rather than pathogen-specific tests, and require validation against active versus latent infection, treatment response and relevant comorbidities before clinical application.

A key consideration is whether these metabolites have been directly associated with disease severity or treatment outcomes in clinical cohorts. To date, the evidence is largely cross-sectional and diagnostic rather than prognostic: plasma lactate, ketone bodies and related glycolytic and TCA-cycle intermediates are consistently elevated in patients with active TB relative to healthy controls [64], and broader metabolomic signatures have been reported to change during anti-tuberculosis therapy, indicating potential value for treatment monitoring. However, robust, prospectively validated associations between these specific metabolites and graded disease severity or hard treatment outcomes, such as time to culture conversion, relapse or mortality, are currently lacking, and existing studies are limited by heterogeneous designs and incomplete adjustment for comorbidities such as HIV and diabetes. These metabolites should therefore be regarded as promising but unvalidated candidates whose prognostic value in TB remains to be established in dedicated longitudinal cohorts.

### 6.2. Muscle and Cachexia Biomarkers for Risk Stratification

Because TB-associated cachexia carries substantial prognostic weight, molecular markers of muscle catabolism are attractive candidates for identifying patients at risk of poor outcomes [48]. Activation of the ubiquitin–proteasome system, indexed by the muscle-specific E3 ubiquitin ligases atrogin-1 and MuRF1, together with markers of mitochondrial dysfunction and oxidative stress, captures the catabolic state that accompanies systemic inflammation in TB. Coupled with anthropometric and functional measures, such molecular indicators could support early identification of accelerated muscle loss and enable stratification for nutritional and host-directed interventions. Longitudinal assessment of these markers may also provide an objective measure of recovery, complementing sputum conversion in evaluating the systemic resolution of disease.

At present, none of these candidate signatures has been prospectively validated as a diagnostic or prognostic biomarker in TB; they should therefore be regarded as hypotheses for future evaluation rather than established clinical tools.

It is important to stress that these candidate markers sit at very different stages of evidence. Some metabolic readouts have already been measured directly in patients with TB: plasma metabolomic studies have reported elevations of lactate, succinate and ketone bodies in TB cohorts relative to healthy controls [64,65], and TCA-cycle remodelling with succinate accumulation has been linked to IL-1β-driven inflammation in human pulmonary TB [65], while numerous host cytokine and inflammatory biosignatures have been evaluated in patient populations for diagnosis and treatment monitoring, though none is yet in routine clinical use [65]. By contrast, the muscle-specific catabolic markers central to the cachexia argument, notably the E3 ubiquitin ligases atrogin-1 and MuRF1 together with molecular indices of muscle mitochondrial dysfunction, have, to our knowledge, not yet been directly reported in skeletal muscle of patients with TB. Their relevance is currently extrapolated from other catabolic and wasting conditions in which they have been measured in human muscle, including cancer cachexia, chronic obstructive pulmonary disease and sepsis [66]. Prospective studies measuring these markers directly in TB patients, and linking them to clinical endpoints, are therefore a priority before any can be considered for clinical application (Table 1).

## 7. Therapeutic Implications: Host-Directed Strategies

The metabolic reprogramming of macrophages during TB presents a promising avenue for therapeutic intervention. By targeting specific metabolic pathways, it is possible to modulate macrophage function, enhance host immunity, and improve bacterial clearance. Strategies to manipulate macrophage metabolism not only have the potential to control the local immune response, but also to mitigate the systemic effects of metabolic dysregulation in TB.

Much of the evidence for these host-directed strategies derives from preclinical models or from clinical experience in other inflammatory and wasting conditions; their efficacy and safety as adjuncts to anti-TB therapy remain to be established in dedicated clinical trials.

### 7.1. Targeting Macrophage Metabolism

Therapeutic interventions aimed at macrophage metabolic pathways focus on modulating key processes such as glycolysis, oxidative phosphorylation, and lipid metabolism. Glycolysis inhibitors, for example, hold promise in rebalancing macrophage activity by suppressing the excessive glycolytic flux observed in M1-polarised macrophages. By reducing the reliance on glycolysis, these inhibitors may decrease the pro-inflammatory cytokine production that drives systemic inflammation and tissue damage, while also limiting the nutrient availability for *Mtb* within the macrophage niche.

Similarly, targeting lipid metabolism offers another pathway for intervention. The formation of lipid droplets in infected macrophages provides a nutrient reservoir for *Mtb*, and strategies that disrupt this lipid storage may reduce bacterial persistence. Modifiers of fatty acid oxidation and cholesterol metabolism have been explored for their ability to alter macrophage polarisation, shifting the balance towards phenotypes that are more effective in bacterial clearance while reducing the inflammatory burden.

The impact of these interventions on host immunity and bacterial clearance is critical. While modulating macrophage metabolism can enhance immune function, it must be carefully balanced to avoid impairing the bactericidal activity of these cells. For example, augmenting mitochondrial metabolism through oxidative phosphorylation enhancers can improve energy production and ROS generation, which are essential for pathogen killing. At the same time, interventions must avoid promoting an immunosuppressive state that could compromise the host’s ability to control infection.

Targeting macrophage metabolism represents a dual opportunity: to directly weaken *Mtb*’s survival strategies and to restore host immune homeostasis. As these approaches are refined, they offer a novel framework for TB therapy, integrating metabolic modulation with traditional antimicrobial treatments to improve outcomes and address systemic complications.

### 7.2. Interventions for Muscle Dysregulation

Muscle wasting is a debilitating complication of TB that significantly impacts patient morbidity and quality of life. Addressing muscle dysregulation requires targeted interventions that combine nutritional, pharmacological, and lifestyle strategies to restore muscle mass, improve mitochondrial function, and counteract the systemic catabolic effects of TB. Nutritional support is foundational in mitigating muscle wasting, with protein-rich diets and supplementation with essential amino acids, such as leucine, helping to stimulate muscle protein synthesis and reduce catabolic processes [67]. Omega-3 fatty acids, with their anti-inflammatory properties, can also modulate the inflammatory environment that drives muscle degradation [68], while micronutrient supplementation, including vitamin D, zinc, and selenium, supports both immune function and muscle metabolism.

Pharmacological interventions complement these nutritional approaches by targeting the molecular pathways involved in muscle wasting. Anabolic agents, such as selective androgen receptor modulators (SARMs) or growth hormone secretagogues, promote muscle protein synthesis and improve physical function [69]. Anti-inflammatory drugs, including TNF-α inhibitors, can reduce cytokine-mediated muscle proteolysis. However, this strategy carries a major and specific risk in the context of TB: TNF-α is essential for granuloma integrity and containment of *Mtb*, and pharmacological TNF-α blockade is a well-recognised cause of TB reactivation and granuloma disorganisation [70]. The risk differs between agents, with monoclonal antibodies generally carrying a higher reactivation risk than soluble-receptor agents. TNF-α inhibition in active or latent TB must therefore be regarded as hazardous rather than straightforwardly beneficial, and any cytokine-directed approach to TB-associated cachexia would require careful weighing of anti-catabolic benefit against the risk of compromising protective host immunity. Additionally, mitochondrial-targeted antioxidants, such as coenzyme Q10 and N-acetylcysteine, alleviate oxidative stress and improve energy production, potentially addressing one of the key metabolic deficits contributing to muscle dysfunction [71].

Exercise also plays a critical role in preserving and recovering muscle in TB patients. Both resistance and endurance training stimulate muscle protein synthesis, enhance mitochondrial biogenesis, and improve overall physical performance. Furthermore, exercise modulates inflammatory signalling pathways, helping to counteract the systemic inflammation associated with TB. Mitochondrial-targeted therapies have emerged as a promising adjunct, with pharmacological agents such as mitophagy inducers and electron transport chain modulators improving mitochondrial energy production and reducing oxidative damage in muscle cells. Mitochondria-specific antioxidants mitigate the effects of reactive oxygen species (ROS), preserving muscle integrity and preventing further degradation.

The integration of nutritional, pharmacological, and exercise-based interventions offers a comprehensive strategy to address muscle dysregulation in TB. These approaches, particularly when combined with mitochondrial-targeted therapies, have the potential to significantly improve muscle health and alleviate one of the most debilitating systemic effects of TB, ultimately enhancing patient outcomes.

### 7.3. Integrated and Host-Directed Approaches

**Figure 5:**
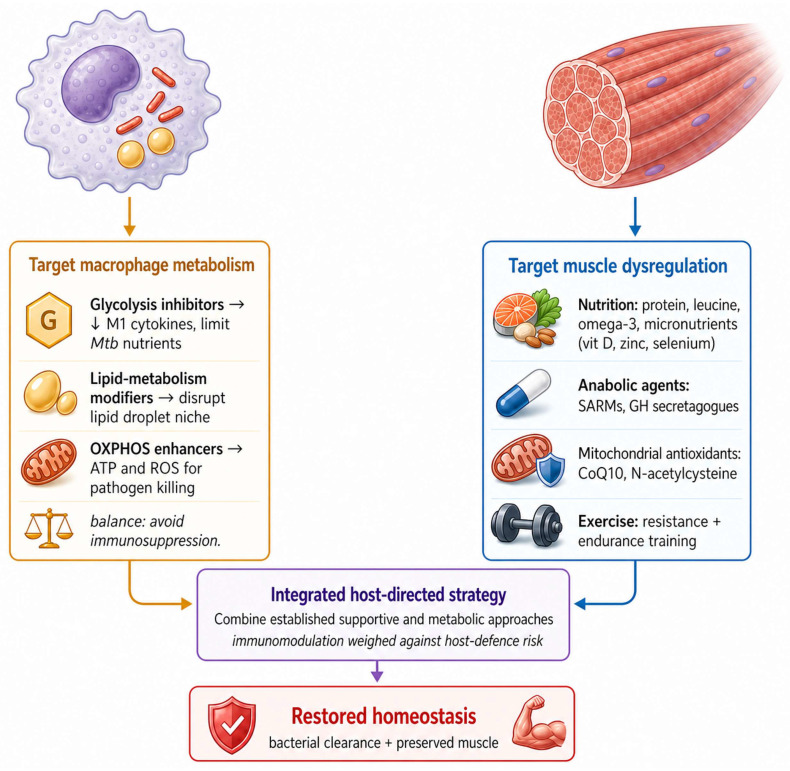
Host-directed therapeutic strategies targeting macrophage metabolism and muscle preservation in TB. Host-directed strategies act on two complementary compartments. Macrophage-metabolism interventions (left) include glycolysis inhibitors, lipid-metabolism modifiers and OXPHOS enhancers, which must be balanced to avoid compromising bactericidal activity. Muscle-directed interventions (right) span nutritional support, anabolic agents, mitochondrial-targeted antioxidants and exercise. These interventions span a spectrum of evidence and risk, from clinically established supportive measures (nutrition, exercise) to experimental metabolic modulators tested largely in preclinical models, and their appropriateness is context-dependent. The arms converge in an integrated strategy combining established supportive and metabolic approaches, with any immunoregulatory intervention weighed against the risk to host defence, aiming to restore homeostasis through bacterial clearance and preserved muscle. Colour coding: amber, macrophage-metabolism interventions; blue, muscle-directed interventions; purple, the integrated strategy; red, the restored-homeostasis outcome. Figure created in Illustrae.co.

Integrated therapeutic strategies targeting both macrophage metabolism and muscle preservation offer a comprehensive approach to addressing the systemic effects of tuberculosis (TB). By recognising the interconnected nature of immune and metabolic dysfunction in TB, these strategies aim to reduce inflammation while supporting muscle health and mitigating the debilitating effects of cachexia. Modulating macrophage metabolism through interventions such as glycolysis inhibitors, lipid metabolism modulators, or mitochondrial enhancers can diminish the production of pro-inflammatory cytokines, including TNF-α, IL-6, and IL-1β, which are central to systemic inflammation and muscle wasting. Simultaneously, therapies focused on muscle preservation, such as anabolic agents, essential amino acid supplementation, and resistance training, can counteract catabolic processes and promote muscle protein synthesis. In principle, pairing anti-inflammatory and anabolic mechanisms could address both the inflammatory and metabolic dimensions of TB-associated muscle wasting. However, such combinations must be approached with caution: direct cytokine blockade, and TNF-α inhibition in particular, carries a recognised risk of TB reactivation because TNF-α is essential for granuloma integrity and containment of *Mtb* [70]. Any integrated strategy incorporating immunomodulation would therefore require careful weighing of anti-catabolic benefit against the risk of compromising protective host immunity, and further research is needed before such approaches could be considered for TB care (Figure 5).

More broadly, the interventions discussed here span a spectrum of evidence and risk. Nutritional support and exercise are clinically established supportive measures; metabolic modulators such as glycolysis inhibitors, lipid metabolism modifiers and mitochondrial-targeted antioxidants remain largely experimental and tested predominantly in preclinical models; and immunomodulatory approaches, particularly TNF-α inhibition, are speculative and potentially hazardous in TB. Their appropriateness is strongly context-dependent, varying with disease stage, bacterial burden and the balance between limiting immunopathology and preserving protective immunity, and rigorous stage-specific clinical evaluation will be required before any can be incorporated into TB care.

## 8. Future Directions

Despite significant advancements in understanding the immunometabolic landscape of tuberculosis (TB), several critical gaps remain that warrant further exploration. One of the most pressing unanswered questions is the precise mechanisms by which macrophage metabolic reprogramming contributes to systemic muscle effects. The metabolic shifts in macrophages, such as enhanced glycolysis or lipid droplet accumulation, likely influence systemic inflammation and muscle metabolism, but the detailed pathways and mediators remain elusive. Another intriguing area for future research is the role of the microbiome in TB-related metabolic dysregulation. The gut microbiota has profound effects on immune and metabolic homeostasis, and its interaction with macrophage activity and muscle health in the context of TB is not yet fully understood [72,73]. Investigating these connections could lead to microbiome-targeted therapies to alleviate systemic complications of TB.

Emerging techniques offer powerful tools to address these knowledge gaps. Single-cell metabolomics and transcriptomics hold immense potential for dissecting tissue-specific interactions and uncovering cell-type-specific responses to TB infection. These high-resolution approaches can reveal how macrophages, muscle cells, and other tissues interact metabolically and immunologically during TB. Additionally, animal models specifically designed to study TB-induced systemic metabolic changes provide a controlled environment to test hypotheses and validate mechanisms. These models enable a deeper understanding of the dynamic interplay between localised infection and systemic metabolic effects, offering a pathway to preclinical therapeutic testing.

Personalised medicine represents a promising frontier in managing systemic complications inherent to TB. Patient-specific metabolic phenotypes, shaped by genetic, environmental, and microbiome factors, likely influence the severity of TB-associated muscle wasting and other metabolic dysregulations. Tailored interventions based on these individual phenotypes could improve outcomes by addressing the unique metabolic and immune profiles of each patient. This approach could encompass personalised nutritional strategies, microbiome-targeted therapies, and metabolically tuned pharmacological interventions.

By addressing these unanswered questions, leveraging emerging techniques, and advancing personalised approaches, future research can uncover new therapeutic opportunities to mitigate the systemic metabolic burden of TB and improve patient care.

## 9. Conclusions

TB exemplifies the complex interplay between immune function and systemic metabolism, with macrophage metabolism playing a central role in driving both local immune responses and systemic muscle dysregulation. The metabolic reprogramming of macrophages during *M. tuberculosis* infection, characterised by enhanced glycolysis, mitochondrial dysfunction, and lipid droplet accumulation, contributes to a pro-inflammatory cytokine milieu. These inflammatory signals, including TNF-α, IL-6, and IL-1β, profoundly disrupt muscle homeostasis, promoting proteolysis, mitochondrial dysfunction, and oxidative stress in muscle tissue. This dynamic interaction not only exacerbates TB-associated cachexia, but also perpetuates a systemic cycle of inflammation and metabolic imbalance.

To address the systemic burden of TB effectively, it is essential to integrate insights from immunology and metabolism into therapeutic strategies. Targeting macrophage metabolic pathways, such as glycolysis and lipid metabolism, offers opportunities to modulate inflammatory responses and enhance bacterial clearance. Simultaneously, interventions aimed at preserving muscle mass, including anabolic therapies, nutritional supplementation, and mitochondrial-targeted treatments, provide a complementary approach to mitigating the effects of TB-associated muscle wasting. Emerging techniques, such as single-cell metabolomics and personalised medicine, hold promise for uncovering novel mechanisms and tailoring interventions to patient-specific metabolic and immune profiles.

By bridging the fields of immunology and metabolism, these integrated approaches have the potential to transform the management of TB, addressing both its localised and systemic manifestations. Such strategies pave the way for innovative therapies that not only improve patient outcomes, but also alleviate the broader systemic impacts of this complex disease.

## Figures and Tables

**Table 1:** Candidate immunometabolic and muscle-derived biomarkers in TB: source, proposed application, and current level of evidence.

Biomarker	Source	Proposed Application	Evidence Level in TB
Lactate	Glycolytic myeloid cells; plasma	Host-response intensity; treatment monitoring	Elevated in TB patients; not validated for severity/outcome
Succinate	Macrophage TCA cycle; plasma	HIF-1α/IL-1β inflammation	Elevated in human TB; not prospectively validated
Ketone bodies (β-hydroxybutyrate)	Systemic energy-stress metabolism	Catabolic/energy-deficit state; immunomodulatory readout	Elevated in TB plasma; prognostic value unestablished
Cytokines (TNF-α, IL-6, IL-1β)	Activated macrophages; plasma	Host-response stratification; treatment monitoring	Measured in TB cohorts; not in routine clinical use
Free fatty acids/lipoprotein profile	Foamy macrophages; systemic lipids	Bacterial persistence and disease activity	Associated with disease activity; requires validation
Atrogin-1 (*FBXO32*)/MuRF1 (*TRIM63*)	Skeletal muscle (ubiquitin-proteasome)	Muscle catabolism; cachexia risk stratification	Not yet reported in TB muscle; extrapolated from cancer cachexia, COPD, sepsis
Mitochondrial dysfunction/oxidative stress indices	Skeletal muscle	Energy deficit; mitochondrial compromise	Mechanistic/extrapolated; not validated in TB muscle
Body composition/fat-free mass	Whole-body/skeletal muscle	Nutritional status and outcome; stratification	Low BMI and lean-mass loss linked to adverse TB outcomes (clinical)

BMI, body mass index; COPD, chronic obstructive pulmonary disease; HIF-1α, hypoxia-inducible factor 1-alpha; IL, interleukin; TCA, tricarboxylic acid; TNF-α, tumour necrosis factor alpha.

## Data Availability

No new data were created or analysed in this study. Data sharing is not applicable to this article.

## Abbreviations

The following abbreviations are used in this manuscript:

TB	Tuberculosis
*Mtb*	*Mycobacterium tuberculosis*
HDT	Host-directed therapy
HIF-1α	Hypoxia-inducible factor 1-alpha
TCA	Tricarboxylic acid
OXPHOS	Oxidative phosphorylation
ROS	Reactive oxygen species
RNS	Reactive nitrogen species
iNOS	Inducible nitric oxide synthase
UPS	Ubiquitin–proteasome system
mTOR	Mechanistic target of rapamycin
PPARγ	Peroxisome proliferator-activated receptor gamma
TNF-α	Tumour necrosis factor alpha
IL	Interleukin
TGF-β	Transforming growth factor beta
SARMs	Selective androgen receptor modulators
